# RETRACTION: Cancer‐Associated Fibroblast‐Derived Exosomal miRNA‐320a Promotes Macrophage M2 Polarization *In Vitro* by Regulating PTEN/PI3Kγ Signaling in Pancreatic Cancer

**DOI:** 10.1155/jo/9845393

**Published:** 2026-07-08

**Authors:** Journal of Oncology

RETRACTION: M. Zhao, A. Zhuang, and Y. Fang, “Cancer‐Associated Fibroblast‐Derived Exosomal miRNA‐320a Promotes Macrophage M2 Polarization *In Vitro* by Regulating PTEN/PI3Kγ Signaling in Pancreatic Cancer,” *Journal of Oncology*, 2022, 9514697, https://doi.org/10.1155/2022/9514697.

The above article, published online on 01 July 2022 in Wiley Online Library (https://onlinelibrary.wiley.com/), has been retracted by agreement between the journal Editor‐in‐Chief, Yoel Klug; and John Wiley & Sons Ltd. The retraction has been agreed due to concerns raised with Figure 6d on PubPeer [1]. Specifically, the p‐AKT bands shown in Figure 6d appear identical to the CPSF‐1/LNCaP bands shown in Figure 3h of [2].

The authors were unresponsive when asked to clarify the concerns and provide the original western blots, and after assessment by the editorial board, the results and conclusions can no longer be considered reliable. The article is therefore retracted.

The authors did not provide a response to the retraction.
